# Mapping the SARS-CoV-2 spike glycoprotein-derived peptidome presented by HLA class II on dendritic cells

**DOI:** 10.1016/j.celrep.2021.109179

**Published:** 2021-05-13

**Authors:** Robert Parker, Thomas Partridge, Catherine Wormald, Rebeca Kawahara, Victoria Stalls, Maria Aggelakopoulou, Jimmy Parker, Rebecca Powell Doherty, Yoanna Ariosa Morejon, Esther Lee, Kevin Saunders, Barton F. Haynes, Priyamvada Acharya, Morten Thaysen-Andersen, Persephone Borrow, Nicola Ternette

**Affiliations:** 1 Centre for Cellular and Molecular Physiology, Nuffield Department of Medicine, University of Oxford, Oxford OX3 7BN, UK; 2 Nuffield Department of Clinical Medicine, University of Oxford, Oxford OX37FZ, UK; 3 Department of Molecular Sciences, Macquarie University, Sydney, NSW 2109, Australia; 4 Duke Human Vaccine Institute, Duke University School of Medicine, Durham, NC 27710, USA; 5 Department of Surgery, Duke University, Durham, NC 27710, USA; 6 Wellbeing Software, Hamilton Court, Oakham Business Park, Mansfield NG185FB, UK; 7 Translational Gastroenterology Unit, Oxford OX3 9DU, UK; 8 The Kennedy Institute of Rheumatology, Oxford OX3 7FY, UK; 9 Biomolecular Discovery Research Centre, Macquarie University, Sydney, NSW 2109, Australia

**Keywords:** SARS-CoV-2, human leukocyte antigen, HLA-II, HLA class II, LC-MS, antigen presentation, dentritic cells, immunopeptidomics, glycoslyation, glycopeptides

## Abstract

Understanding and eliciting protective immune responses to severe acute respiratory syndrome coronavirus 2 (SARS-CoV-2) is an urgent priority. To facilitate these objectives, we profile the repertoire of human leukocyte antigen class II (HLA-II)-bound peptides presented by HLA-DR diverse monocyte-derived dendritic cells pulsed with SARS-CoV-2 spike (S) protein. We identify 209 unique HLA-II-bound peptide sequences, many forming nested sets, which map to sites throughout S including glycosylated regions. Comparison of the glycosylation profile of the S protein to that of the HLA-II-bound S peptides reveals substantial trimming of glycan residues on the latter, likely induced during antigen processing. Our data also highlight the receptor-binding motif in S1 as a HLA-DR-binding peptide-rich region and identify S2-derived peptides with potential for targeting by cross-protective vaccine-elicited responses. Results from this study will aid analysis of CD4^+^ T cell responses in infected individuals and vaccine recipients and have application in next-generation vaccine design.

## Highlights

• DC-presented HLA-II-bound peptidome of SARS-CoV-2 spike glycoprotein
• Spike glycans are truncated during glycopeptide processing and presentation
• The spike receptor binding motif is a HLA-II-binding peptide-rich region
• Concordance between the immunopeptidome and T cell immunogenicity data reported elsewhere

## Graphical abstract

**Figure:**
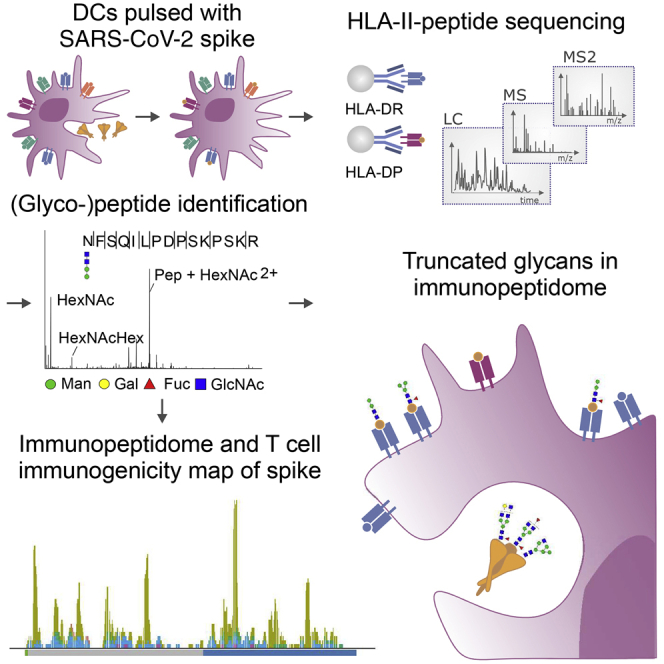

## Teaser

Parker et al. map the HLA-II-bound peptides and glycopeptides presented by SARS-CoV-2 spike protein-pulsed monocyte-derived dendritic cells. They observe that complex glycans on the spike immunogen are trimmed during antigen processing, revealing a signature for HLA-II presentation, and highlight congruence between the HLA-II-bound peptides identified and T cell epitopes.

## Introduction

Severe acute respiratory syndrome coronavirus 2 (SARS-CoV-2) is a novel beta-coronavirus that first emerged as a human pathogen in the Hubei province of China in late 2019 and is the etiologic agent of coronavirus disease 2019 (COVID-19). Although SARS-CoV-2 infection is frequently asymptomatic or results in only mild illness, ∼20% of symptomatically infected individuals progress to develop severe pneumonia, acute respiratory distress syndrome, and/or sepsis, which can be fatal. By the 31^st^ of January 2021 102,139,771 cases and 2,211,762 deaths had been reported worldwide (WHO, 2021b). The rapid global spread of SARS-CoV-2 and the resulting pandemic have placed tremendous pressure on healthcare services, had a huge societal impact, and profoundly damaged the global economy, prompting an urgent need for effective vaccination campaigns to prevent further spread of infection and avert disease development (WHO, 2021b).

Immune correlates of protection against SARS-CoV-2 infection and progression to severe disease are still not well understood, although infection was found to induce at least short-term protective immunity in a SARS-CoV-2 non-human primate (NHP) infection model, indicating that immune responses are capable of mediating protection (Chandrashekar et al., 2020). Passively transferred neutralizing antibodies (nAbs) protect against SARS-CoV-2 infection in small animal models, and convalescent sera have been shown to be effective in the treatment of severe disease, suggesting the utility of nAb induction by vaccines (Brouwer et al., 2020; Zost et al., 2020; Liu et al., 2020). Notably, the four seasonal common cold-causing human coronaviruses, the zoonotic Middle East respiratory syndrome (MERS), and SARS coronaviruses typically elicit poorly sustained nAb responses, putatively enabling subsequent re-infection (Kellam and Barclay, 2020). However, somewhat more durable T cell responses are induced, which in animal models can prevent development of severe disease on challenge, providing a rationale for vaccine-mediated induction of T cell as well as nAb responses (Gallais et al., 2020; Libraty et al., 2007; Yang et al., 2006; Zhao et al., 2010). More than 230 candidate SARS-CoV-2 vaccines are now in preclinical development or clinical trials (WHO, 2021a), and several vaccines are in widespread clinical use (Creech et al., 2021).

The SARS-CoV-2 spike (S) glycoprotein (comprising S1 and S2 subunits) is the primary target of vaccine development efforts. Homotrimers of the transmembrane S protein on the virion surface mediate virion attachment and entry into host cells, making S a key target for nAbs (Letko et al., 2020). S is also highly immunogenic for T cells, with many studies suggesting that although infected individuals mount CD4^+^ and CD8^+^ T cell responses to epitopes throughout the viral proteome, S is often at the top of the antigenic hierarchy (Grifoni et al., 2020b; Altmann and Boyton, 2020; Weiskopf et al., 2020). The relative roles of CD4^+^ and CD8^+^ T cells in disease control or pathogenesis and impact of their protein and epitope specificity are unknown, but given the importance of CD4^+^ T cells (particularly CD4^+^ T follicular helper [Tfh) cells) in providing help for antibody responses (Crotty, 2019), and the correlation of memory B cell/nAb responses to S with circulating CD4^+^ Tfh responses in recovered COVID patients (Juno et al., 2020), induction of potent Tfh cell responses to the S protein is likely to be crucial for the success of nAb-inducing vaccines.

CD4^+^ T cells are initially activated in response to recognition of specific peptides presented with major histocompatibility complex class II (MHC-II) molecules on professional antigen presenting cells such as dendritic cells (DCs) (Roche and Furuta, 2015). The human MHC-II region encodes polymorphic human leukocyte antigen (*HLA*)-*DRA/DRB1*, -*DRA/DRB3*, -*DRA/DRB4*, -*DRA/DRB5*, -*DPA1/DPB1* and -*DQA1/DQB1* molecules, with *HLA-DRA/DRB1* being expressed at the highest and *HLA-DQA1/DQB1* at the lowest levels (Robinson et al., 2015; Yamamoto et al., 2020). HLA-II polymorphisms dictate the repertoire of peptides presented for CD4^+^ T cell recognition and shape the response elicited, which can influence the outcome of infection or vaccination (Unanue et al., 2016). Here, we defined SARS-CoV-2 S-derived peptides presented with diverse HLA-II alleles on DCs to facilitate analysis of pre-existing, post-infection or vaccine-elicited S-specific CD4^+^ T(fh) cell responses and their roles in protection, pathogenesis, and prevention of re-infection.

## Results

### Approach for analysis of SARS-CoV-2 S HLA-II presentation by MDDCs

**Figure 1:**
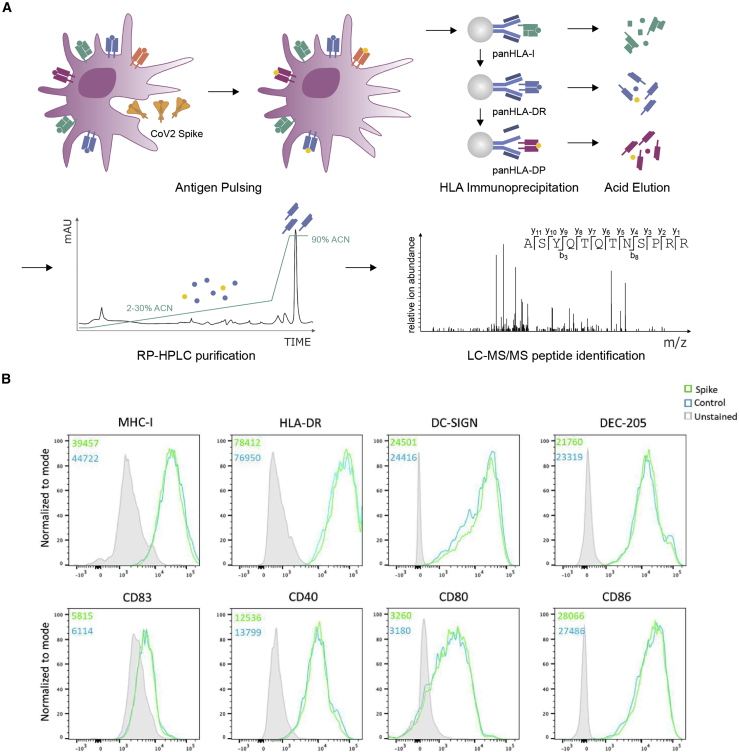
Generation of MDDCs for HLA-II immunopeptidomic profiling (A) Schematic depicting the workflow used to generate the recombinant S protein pulsed MDDC immunopeptidome. MDDCs were pulsed with S or control protein for 18 h, and sequential HLA IPs were carried out as indicated. Peptides were eluted from HLA molecules, purified by preparative HPLC, and subjected to a LC-MS/MS identification workflow. (B) MDDCs were harvested following overnight protein pulse and stained with antibodies to identify DCs and assess their expression of HLA-I, HLA-DR, DC-SIGN, DEC-205, CD83, CD40, CD80, and CD86 and then analyzed by flow cytometry. Histogram plots are gated on live CD11c^+^CD4^+^ DCs and show expression of each indicated marker on cells treated with S or control protein. Unstained cells are shown in filled gray histograms. Inset numbers indicate mean fluorescence intensity for each sample. See also Figure S2.

To identify peptides in the SARS-CoV-2 S protein with potential for targeting by CD4^+^ T cell responses, a mass-spectrometry-based immunopeptidome profiling approach was employed to define peptides presented by HLA-II on DCs, antigen presenting cells that play a key role in *in vivo* CD4^+^ T cell priming (Figure 1A). MDDCs were generated from 5 HLA-DRB1-heterozygous donors, selected to enable profiling of peptides presented with a total of 9 different HLA-DRB1 alleles, 5 different HLA-DRB3/4/5 alleles, and 7 distinct HLA-DPB1 alleles (Table 1). MDDCs from each donor were pulsed with a recombinant SARS-CoV-2 S protein vaccine immunogen candidate (Henderson et al., 2020) produced in a mammalian cell expression system (Figure S1), or a recombinant viral glycoprotein from an unrelated virus that had been produced in the same way to provide a negative control dataset, and incubated for 18 h to allow antigen uptake, processing, and presentation. Flow cytometry analysis indicated that, as anticipated, the CD11c^+^ MDDC population had robust expression of HLA-I and HLA-DR, expressed high levels of the lectin-type receptors DC-SIGN and DEC-205 and had a relatively immature phenotype, expressing low levels of the DC maturation marker CD83 and moderate levels of the costimulatory molecules CD40, CD80, and CD86. Notably, no difference was observed in the phenotype of SARS-CoV-2 S protein and control protein-pulsed MDDCs, indicating that the S protein had not altered the DC maturation state or HLA expression levels (Figure 1B; Figure S2).

**Table 1:** Donor HLA-DRB1, -DPB1, and -DQB1 alleles

Donor	DRB1		DRB3	DRB4	DRB5	DPB1		DQB1	
C2	13:03	15:01	01:01	nf	01:01	03:01	04:02	06:02	03:09
C459	04:01	12:01	02:02	01:03	nf	04:01	04:02	03:01	03:01
C460	04:01	14:54	02:02	01:03	nf	15:01	10:01	05:03	03:01
C491	01:02	11:01	02:02	nf	nf	04:01	14:01	03:01	05:01
C493	07:01	03:01	01:01	01:01	nf	04:01	01:01	02:02	02:01

nf, not found.

### Immunopeptidomic profiling of HLA-II-associated peptides presented by MDDCs

**Figure 2:**
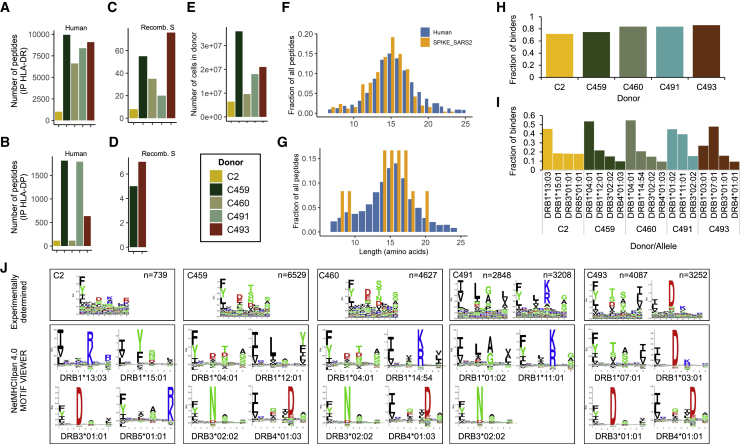
Immunopeptidomic profiling of HLA-DR and HLA-DP-bound peptides presented by S-pulsed MDDCs (A and B) Number of unique peptide sequences identified in each HLA-DR (A) or HLA-DP (B) immunoprecipitation sample. (C and D) Number of unique peptide sequences that map to S identified in the HLA-DR (C) or HLA-DP (D) samples. (E) Total number of MDDCs harvested from each donor. (F and G) Length distribution of peptide sequences identified as mapping to human proteins (blue) or S protein (orange). (H) Proportion of peptide sequences predicted to bind to each donor’s HLA-DR and -DP alleles by NetMHCIIpan 4.0. (I) Proportion of total predicted HLA-DR binders stratified by allele for each donor. (J) All 12–20 mers in each sample were clustered using the online (unsupervised) GibbsCluster algorithm. Each cluster is represented by a sequence logo, which corresponds to at least one of the HLA-DRB alleles expressed by the donor MDDCs. Amino acids are represented by their single letter code; the more frequently an amino acid occurs a position within peptides, the larger the letter is displayed. The number (n) of peptides within each cluster is indicated along with the number of outlier peptides removed, and clusters are presented with the specific sequence motifs for donor DR alleles as reported by NetMHCIIpan 4.0. See also Figures S4 and S5 and Table S1.

Protein-pulsed MDDCs were lysed and sequential immunoprecipitations performed with a pan-HLA-I-specific antibody (W6/32) for depletion of HLA-I complexes, followed by serial pan-HLA-DR (L243) and pan-HLA-DP (B721) immunoprecipitations for enrichment of HLA-DR- and HLA-DP-peptide complexes. After peptide elution and sequencing by tandem mass spectrometry, a total of 27,081 unique HLA-DR- and 2,801 HLA-DP-associated peptide sequences were identified at 1% false discovery rate (FDR), of which 147 (HLA-DR) and 12 (HLA-DP) mapped to the S protein (Figures 2A–2D; Table S1). None of these peptides were identified in the control protein-pulsed MDDC samples (data not shown), consistent with derivation from the S protein antigen. The total number of identified peptides varied in each donor and was influenced by starting cell numbers (Figures 2A–2E). The overall peptide length distributions were highly characteristic of HLA-II-associated peptides, with a median amino acid length of 15 for both human and S peptides (Figures 2F and 2G). The immunopeptidome is dependent on the genotype and abundance of the HLA alleles expressed in an individual. To validate the sequences reported here, we performed binding predictions using NetMHCIIpan 4.0 and found that 72%–86% of the peptide sequences identified had a high predicted binding affinity for one or more of the relevant donor’s HLA-DR or -DP alleles (Figure 2H). When stratified by the HLA-DRB allele to which each peptide exhibited the highest predicted binding affinity, the majority (64%–85%) of peptides were predicted to be bound by DRB1 alleles, with 15%–35% of peptides predicted to be bound by DRB3/4/5 alleles (Figure 2I). This was further visualized in an unsupervised Gibbs clustering analysis, which revealed distinct sequence motifs characteristic of at least one of the donor’s HLA-DRB alleles (Figure 2J).

In order to assess the relative protein abundance of DR alleles in the individual donors, we performed a proteomic analysis of the HLA-DR immunoprecipitate. The relative abundance of the relevant DRB protein in the HLA-DR immunoprecipitate as determined by quantitative proteomics (Figure S3A) showed a strong positive correlation with the proportion of peptides predicted to bind with highest rank scores to each HLA-DRB allele (R2 = 0.915, Figure S3B). These results indicate that the relative HLA-DRB protein expression levels are an important determinant of the HLA-DR-associated peptide sequence repertoire.

Prior to purifying class II complexes, we also purified HLA-I ligands and identified 29,309 self-peptides. No MDDC HLA-I cross-presentation of the pulsed S protein was detected (data not shown).

### Multiple regions of S are presented by HLA-DR in a genotype-dependent manner

**Figure 3:**
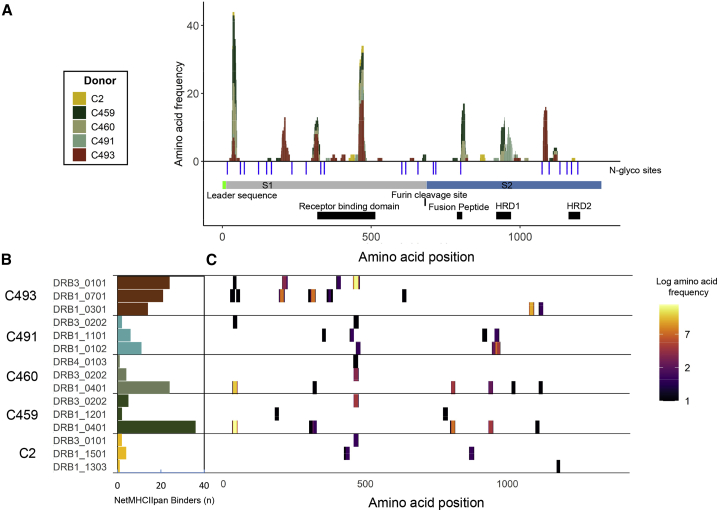
Distribution of HLA-II-presented regions within the S glycoprotein (A) Stacked histogram of positional amino acid frequency in S for all peptide sequences identified in the immunopeptidome stratified and colored by donor. Horizontal bars depict the S1/2 regions, and blue vertical lines indicate N-linked glycosylation sites identified in the purified protein detected by digestion with PNGase F in the presence of heavy water, which leads to a detectable specific mass shift of 2 Da when an N-linked glycan has been removed. The furin cleavage site, receptor binding domain, fusion peptide, and heptad repeat domains (HRD) 1 and 2 are depicted as black boxes. (B) Number of S peptide sequences predicted to bind to each donor HLA-DR allele by NetMHCIIpan 4.0. C. Position of the identified DR-bound peptides across the S sequence, depicted as a heatmap of amino acid coverage and stratified by the donor and predicted DR allele of origin on the y axis. See also Figures S4 and S5 and Tables S1 and S2.

Characteristic of HLA-II-bound peptides, many of the S peptides identified (Table S1) formed distinctive nested sets around a common core. Two of the identified peptides originated from regions that were altered to assist recombinant protein expression and purification (Figure S1C). The location of the identified S peptides in the context of protein region and domain structure and relative frequency with which particular sites are presented in each donor is summarized in Figure 3A. Several “hotspots” from which a large number of unique peptides (typically different members of a nested set) are presented in multiple donors are apparent across the length of the full S protein, and two regions of S, spanning amino acids 24–49 and 457–485, particularly stand out as the sites from which the highest number of unique HLA-II-bound peptides were derived.

To explore the contribution of individual HLA-DR alleles to presentation of the S protein, we investigated the likely allele to which each peptide was bound using HLA-II-binding prediction (NetMHCIIpan 4.0) (Figures 3B and 3C). 77%–95% of the HLA-DR-bound S peptide sequences identified in each donor were predicted to bind one or more of the donor’s HLA-DR alleles (Figure S4A). This stratification demonstrated a within-patient allele usage bias in S presentation that in most donors mirrored the previously observed bias in the percentage of all peptides presented by individual alleles (Figures 3B and 2I). For example, the majority of both self and S peptides were presented with the DRB1^∗^04:01 allele in donor C459 and C460 MDDCs, although in donor C493 the proportion of S peptides showing the highest predicted affinity of binding to DRB3^∗^01:01 was higher than that observed for self-peptides and exceeded values for this donor’s DRB1 alleles (Figures 2I and 3B). A strong correlation was observed between the DRB1^∗^04:01-presented peptide profile of donors C460 and C459, who both expressed this allele (R = 0.99), and both donors shared by far the largest number of identical peptide sequences (23) found in any pairwise comparison (Figures S4B, S4C, and S5).

### HLA-II-bound S peptides with N-linked glycosylation predominantly bear truncated paucimannose glycans

To determine the glycosylation status of the S protein immunogen used in this study, a proteomic approach was used to map the N-linked glycosylation sites, involving *in vitro* digestion of the recombinant S protein, trimming of glycans from the generated peptides with PNGaseF in the presence of heavy water (H_2_^18^O), and peptide characterization by mass spectrometry (Liu et al., 2010). This analysis revealed that the 22 N-linked glycosylation sites previously described in S (Watanabe et al., 2020) were occupied in the S protein employed here (Table S2). Notably, regions of the S protein containing glycosites were devoid of peptides identified in our initial analysis of the HLA-II-bound peptidome, raising the question of whether S-derived glycopeptides were also presented by MDDCs (Figure 3A).

**Figure 4:**
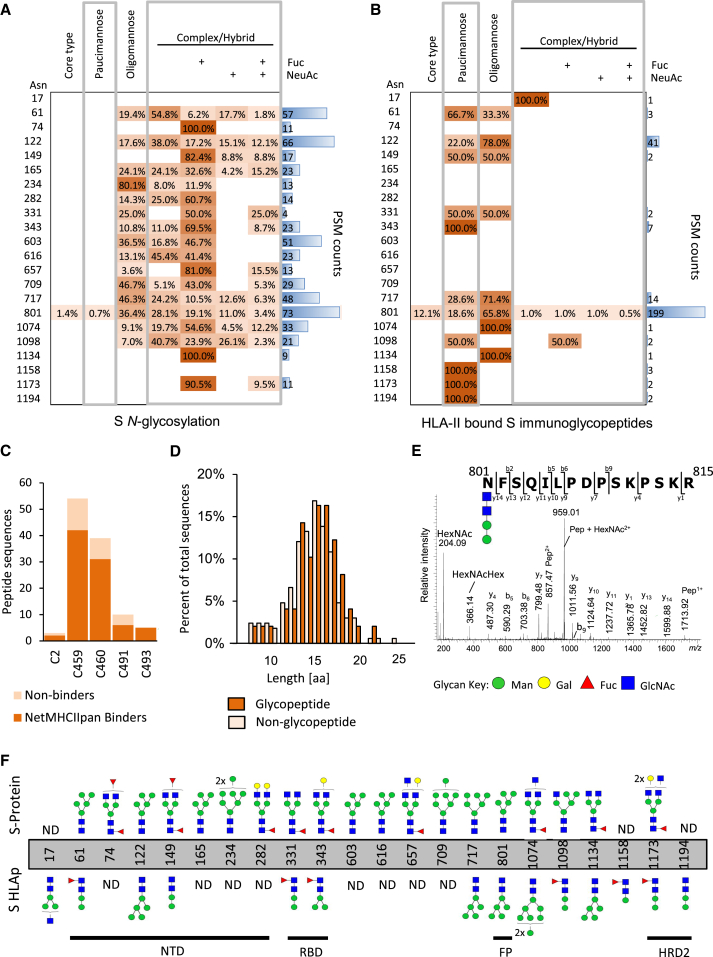
Glycosylation site and glycopeptide analysis of recombinant S protein and HLA-II-bound S peptides (A and B) Table showing distribution of glycans identified at each glycosylation site grouped according to the main *N*-glycan types based on intact glycopeptide analysis. (A) glycopeptides generated by *in vitro* digestion of the recombinant S protein and (B) HLA-II-bound S glycopeptides detected in the immunopeptidome of S-pulsed MDDCs. The heatmap colors indicate the relative frequency of each glycan composition present. The total number of peptide spectral matches (PSM) is reported (blue bars). (C) Donor-specific overview of the number of glycosylated peptide sequences and proportion predicted to bind to the donor HLA-DR allele profile. (D) Histogram illustrating the amino acid length distribution of glycopeptides and non-modified peptide sequences of S. (E) Example of an annotated fragment spectrum of an HLA-II-bound peptide carrying a paucimannosidic-type *N*-glycan in position N801. (F) Depiction of the most abundant glycan identified at each *N*-glycosylation sites of the S polypeptide chain prior to MDDC pulsing (top) and of the HLA-II-bound S peptides (bottom). The main domains of S are indicated. Man, mannose; Gal, galactose; Fuc, fucose; NeuAc, *N*-acetylneuraminic acid; GlcNac, *N*-Acetylglucosamine G. See also Tables S2, S3, and S4.

To enable glycopeptide analysis, non-PNGaseF-treated S digests were analyzed using a well-established glycoproteomics strategy (Alves et al., 2017). Using this approach, glycopeptides at 19 sites of S were identified to carry oligomannosidic and complex/hybrid-type *N*-glycans (Figure 4A; Table S3). Most sites displayed extensive glycan microheterogeneity arising from differences in both glycan types and structural features including terminal sialylation and fucosylation, in agreement with the known site-specific glycosylation of S (Watanabe et al., 2020).

Next, we applied the site-specific glycopeptide methodology to the mass spectra acquired from samples eluted from HLA-II (Figure 4B). 80 distinct glycopeptide forms mapping to S were identified; the majority of these (76) were derived from the HLA-DR-bound immunopeptidome (Table S4). These glycopeptide forms mapped to 52 unique peptide sequences that typically formed nested sets, were predominantly observed in datasets generated from donors C459 and C460 (where the highest number of unique HLA-DR-bound non-glycosylated peptides were also detected), and had a similar length distribution to S-derived non-glycopeptides (Figures 4C and 4D). 75% (66%–100%) of all glycopeptide sequences were predicted (using NetMHCIIpan 4.0) to bind to one or more of the donor’s HLA-DR alleles (Figure 4C). The largest nested set consisted of glycopeptides from donor C459/C460 MDDCs that mapped to position N801 located directly in the fusion peptide (FP, 788–806), a highly conserved region that facilitates membrane fusion during viral entry (Figures 4E and 4F). In total, we identified HLA-II-bound glycopeptides bearing glycans derived from 14 of the N-linked glycosylation sites in S (Figure 4F). HLA-II-bound peptides carried predominantly short paucimannosidic-type *N*-glycans while S carried oligomannosidic- and GlcNAc-capped complex-type *N*-glycan structures at these sites (Figures 4B and 4F). The paucimannosylation of the HLA-II-bound peptides comprised both core-fucosylated (M1F, M2F, and M3F i.e., Man_1–3_GlcNAc_2_Fuc_1_) and a fucosylated (M2, Man_2_GlcNAc_2_) species, as supported by fragment spectra analysis (Figure 4E).

In addition, utilizing a global post-translational modification (PTM) peptide identification methodology, we identified peptides containing the other most common peptide modifications (bar glycans) in the S immunopeptidome. Modifications of cysteine (glutathione disulfide, cysteine oxidation, and cysteinylation) were more commonly observed in S compared to HLA-II-bound peptides of human origin, while modifications of other amino acids (deamidation of glutamine and asparagine, oxidation and per-oxidation of methionine, and conversion of N-terminal glutamine to pyroglutamic acid) were less commonly observed in S (Figure S6A). A total of 27 peptides with modified cysteine residues were identified that mapped to 6 positions in S (Figure S6B). All peptides contained a single modified cysteine residue known be involved in forming a disulphide bond in the tertiary structure of S (Walls et al., 2020).

### Peptides derived from the RBM of the SARS-CoV-2 S protein are presented in all donors

**Figure 5:**
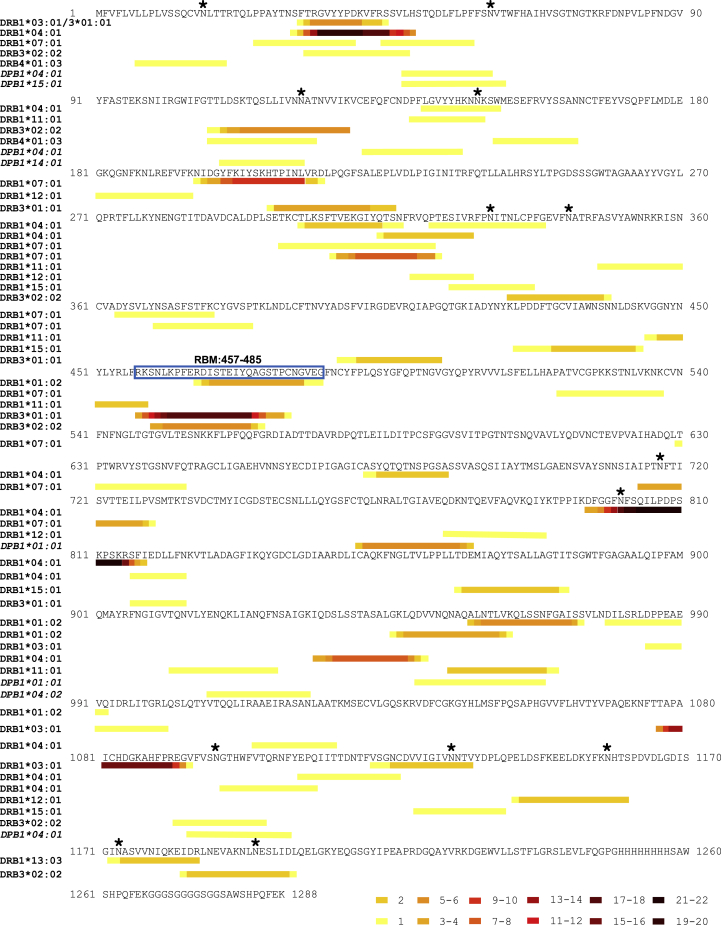
HLA-II peptide mapping across the S protein and RBM A map illustrating the location in the SARS-CoV-2 protein sequence of all the S-derived peptides of 9–25 amino acids in length identified in the HLA-II-bound immunopeptidome of S-pulsed MDDCs. HLA-DR-bound peptides are grouped according to the HLA-DR allele to which they had the highest predicted binding affinity (as determined using NetMHCIIpan 4.0), and HLA-DP-bound peptides are grouped by donor HLA-DPB1 type. Within each group, nested peptide sets are indicated in heatmap form, where the color represents the frequency of each amino acid position within the peptide group. Where a single peptide amino acid sequence was identified multiple times with different sequence modifications the unique amino acid sequence was included only once in positional frequency calculations. Glycosylation sites in S identified in the Byonic analysis of the immunopeptidome are indicated with an asterisk, and the nested set covering the RBM is boxed.

Altogether, a total of 209 unique HLA-II-bound peptides (differing in amino acid sequence) derived from the SARS-CoV-2 spike protein were detected in this study. The locations and HLA-II alleles putatively presenting these peptides (typically members of large, nested sets) are summarized in Figure 5. Partly overlapping nested sets of peptides predicted to be presented by distinct HLA-DR alleles in different donors were identified in several regions of S1, and also in the more sequence-conserved S2 protein (Lei and Zhang, 2020), highlighting the potential for broad HLA-II presentation of multiple regions of this key virion glycoprotein.

**Figure 6:**
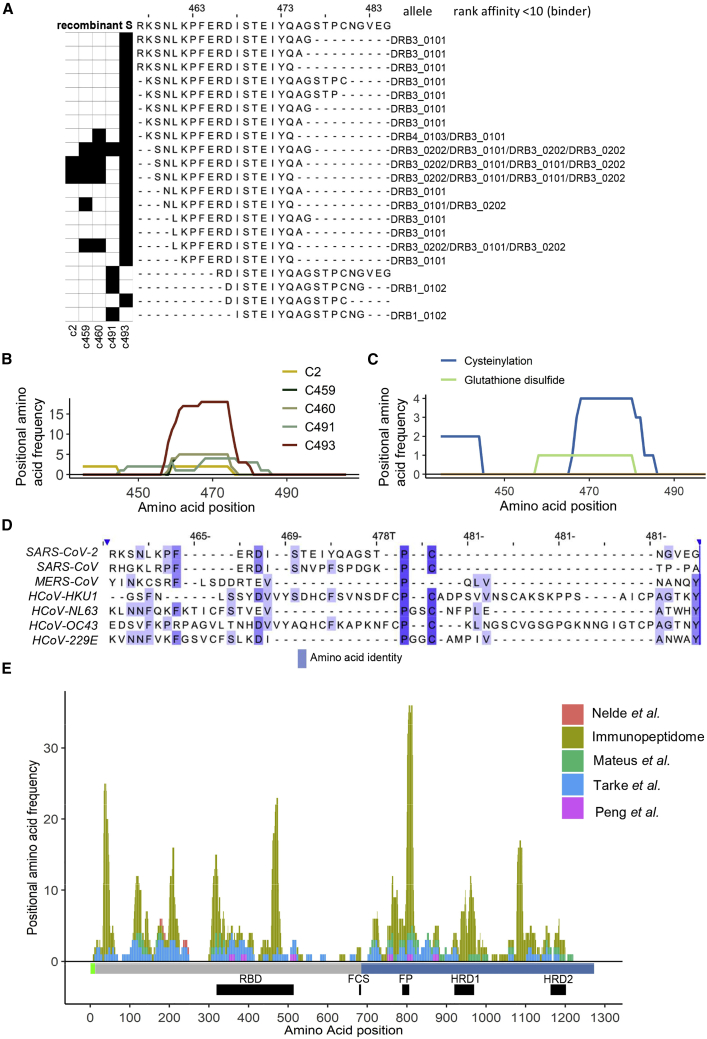
A nested set of unique peptides in the immunopeptidome map to positions 457–485 in the RBD of the full-length S protein (A) The black squares on the left indicate the donor(s) in which each peptide was identified. On the right of each peptide sequence, the HLA-DR allele to which it showed the highest predicted affinity of binding (NetMHCIIpan4.0), and the regarding rank score is indicated. (B) Line plots showing the frequency with which each amino acid position was represented by a peptide identification across the RBD of the S protein in each donor. (C) Line plots showing the frequency with which each amino acid position was represented by a cysteine-modified peptide identification (cysteinylated peptides and glutathione-modified peptides) across the RBD of the S protein. (D) Alignment of the S protein sequences of the SARS-CoV-2, SARS-CoV, MERS-CoV, and 4 endemic human coronaviruses (obtained from Uniprot). Positions relative to the RBD amino acids 457–485 in S are shown. The color indicates the degree of amino acid identify. (E) Stacked histogram plot showing the peptides identified as being recognized by CD4^+^ T cells in each of the four T cell epitope mapping studies and those in the immunopeptidome across the S protein sequence. The height of each bar represents the frequency at which each amino acid was detected in the relevant dataset. The domain structure for S is indicated below the plot as horizontal bars (S1/2 regions: gray/blue); and the furin cleavage site (FCS), receptor binding domain (RBD), fusion peptide (FP), and heptad repeat domains (HRD) 1 and 2 are depicted as black boxes.

Within the receptor binding domain (RBD) of S1, the receptor binding motif (RBM), an extended insert that contains the contact points with the receptor ACE2 (Lan et al., 2020) and is an important nAb target, contained 2 nested sets of peptides predicted to be presented by 3 different HLA-DR alleles (Figure 5). In the donors analyzed, a total of 21 unique peptide sequences derived from this region were identified altogether (Figures 6A and 6B), and versions of some of these post-translationally modified at residues C480 and C432 were also detected (Figure 6C). At least one peptide within this region was found to be presented in every donor studied. Interestingly, a particularly large nested peptide set, presented in all donors, was predicted to be bound by HLA-DR3, highlighting a potentially central role of this gene in presenting antigenic peptides derived from the S RBD domain (Figure 3B and 5).

To gain insight into the sequence conservation of this RBD region in other coronaviruses infecting humans, S protein sequences from SARS-CoV-2, SARS-CoV, and MERS-CoV (the other beta-coronaviruses that have caused epidemics in humans in the past two decades) and the endemic human coronaviruses 229E, NL63, OC43, and HKU1 were aligned (Figure 6D). Although this region of the SARS-CoV-2 S protein showed some sequence similarity with the equivalent region of the SARS-CoV S protein, this is an indel-rich region of S that was much less well conserved in the other coronaviruses examined. However, although residues that are likely to constitute key anchors in the core regions of the nested peptide sets predicted to bind to particular HLA-DRA/DRB1 molecules (SARS-CoV-2 F464 and S469, which match anchor residue preferences in DRB1^∗^04:01-, 07:01-, 13:03-, and 15:01-binding peptides; F464 and D467, which match preferred anchors of DRB1^∗^03:01-binding peptides; and I472 and S477, which match those of DRB1^∗^01:01-binding peptides) are not well conserved or not appropriately positioned relative to one another in all coronaviruses, there appears to be some potential for HLA-II-binding peptides to be generated from this region of other human coronavirus S protein sequences.

To understand the relationship between our HLA-II-bound spike peptide dataset and the epitopes in the spike protein targeted by CD4^+^ T cell responses, we assessed the overlap between the HLA-II-bound S peptide sequences identified in our study and S peptides to which CD4^+^ T cell responses were detected in four recent T cell epitope mapping studies (Mateus et al., 2020; Nelde et al., 2021; Peng et al., 2020; Tarke et al., 2021). Differences in the sensitivity of the methods employed for CD4^+^ T cell response evaluation and the number of spike peptides to which responses were tested (three studies screened overlapping peptides spanning the entire spike protein sequence, but Nelde et al. (2021) tested just two spike peptides predicted to bind commonly expressed HLA-DR alleles) impacted on the number of peptides to which responses were identified by each group of authors (Table S5; Figure S7). There were also inter-study differences in the sequences defined as T cell epitopes, as these studies were performed in HLA-diverse subjects, and whereas three groups of authors employed T cells from SARS-CoV-2 convalescent patients for peptide screening, Mateus et al. (2020) focused on identifying pre-existing cross-reactive T cell responses to SARS-CoV-2 and so performed their peptide screening in individuals exposed only to seasonal coronaviruses. Together, the 93 HLA-II restricted peptides to which CD4^+^ T cell responses were detected in one or more of these publications spanned 57% of the SARS-CoV-2 S protein sequence used in this study and 74% of the immunopeptidome sequence, indicative of substantial enrichment in the latter. Moreover, 67% of the amino acids contained within T cell targeted peptides were located in peptides in the immunopeptidome (Figure 6E; Figure S7). Given that T cell response screening was performed with overlapping sets of long peptides and epitopes were not precisely defined within these, and the more distal regions of the HLA-II associated peptides defined by our immunopeptidome profiling strategy may not be required for T cell recognition, the amino acid overlap between the T cell-recognized and HLA-II-bound peptide sequences provides an under-estimate of the concordance between the epitopes targeted by CD4^+^ T cell responses and the repertoire of HLA-II-bound peptides. Overall, the extensive overlap observed between these datasets (Figure 6E) confirms the utility of our approach of profiling the peptides presented with HLA-II on antigen-pulsed dendritic cells for identification of peptides with potential for *in vivo* T cell recognition. Moreover, as considered further in the discussion, this analysis highlights sites within the more conserved SARS-CoV-2 S2 protein that are not commonly recognized by CD4^+^ T cells in infected individuals but have potential for targeting by vaccines aiming to elicit responses with greater inter-coronavirus cross-reactivity.

## Discussion

Concurrently with the design and clinical evaluation of candidate immunogens in the race to develop and improve vaccines with prophylactic efficacy against SARS-CoV-2 infection and associated disease, there is an urgent need to define T cell epitopes to facilitate analysis of the contribution of T cell responses to protection and pathogenesis in infected individuals and monitoring of immune responses elicited in human vaccine trials (Altmann and Boyton, 2020). As the SARS-CoV-2 S protein is the major target on the virus for neutralizing antibodies (Rogers et al., 2020; Brouwer et al., 2020; Zost et al., 2020; Hansen et al., 2020; Liu et al., 2020; Cao et al., 2020; Casadevall and Pirofski, 2020; Mair-Jenkins et al., 2015) and has also been shown to be highly immunogenic for T cell responses in infected individuals (Grifoni et al., 2020b; Altmann and Boyton, 2020; Braun et al., 2020; Weiskopf et al., 2020) a high proportion of the SARS-CoV-2 vaccines in pre-clinical and clinical development focus on eliciting immune responses to this protein. In this study, we have defined SARS-CoV-2 S-derived peptides presented by DCs, a cell type crucial for induction of immune responses during infection and after vaccination (Unanue et al., 2016), following uptake and processing of exogenously acquired S protein. We identify a total of 209 unique HLA-II-bound peptide sequences, including members of 27 nested peptide sets, demonstrating presentation of both glycopeptides and peptides with other post-translational modifications. Notably, our analysis reveals that nested peptide sets derived from a region of the RBD that overlaps with the RBM were presented in all of the HLA-DR-diverse donors studied here, highlighting this as a region of the SARS-CoV-2 S protein that could putatively be targeted by T cell responses in multiple individuals. The peptides identified in this study provide an important resource that will expedite (1) exploration of pre-existing T cell responses to other coronaviruses, (2) cross-comparison of responses elicited by different vaccine immunogens and platforms, and (3) design of next-generation vaccines tailored to elicit enhanced responses to nAb epitopes, or focus T cell responses on selected epitopes.

The importance of defining SARS-CoV-2-derived peptides presented by diverse HLA alleles is illustrated by the plethora of recent efforts to employ *in silico* approaches to predict putative T cell epitopes in SARS-CoV-2 proteins (Grifoni et al., 2020a; Sohail et al., 2021). Our data give key insight into the repertoire of peptides that are in fact presented with HLA-II when exogenous S protein is internalized and processed by DCs, mimicking a scenario occurring as T cell responses are induced during natural infection or following vaccination with protein immunogens or vaccine vectors that drive protein expression in cell types other than DCs. Whether these peptide profiles are also representative of those presented on DCs in which the S proteins is endogenously expressed (e.g., as may occur in some DCs following antigen delivery with viral vectored or nucleic-acid-based vaccine platforms), which may lead to antigen processing and peptide association with HLA-II in different intracellular compartments, remains to be determined (Roche and Furuta, 2015). Furthermore, although no *in vitro* cross-presentation of S on HLA-I by MDDCs was detected in this study, vaccine platforms that drive intracellular antigen expression would be expected to result in HLA-I presentation of S-derived peptides, promoting induction of CD8^+^ T cell responses (Jackson et al., 2020; van Doremalen et al., 2020).

77%–95% of the peptides we identified were predicted to bind to at least one of the donor’s HLA-DR/DP alleles, and the majority (54%–89%) bound to DRB1, which was consistent with the higher expression and more dominant antigen presenting role of HLA-DRB1 versus HLA-DRB3/4/5 molecules, as observed previously (Juno et al., 2020). While HLA-II binding predictions suggested that the two HLA-DRB1 alleles expressed in some donors made roughly equal contributions to the repertoire of unique peptides presented, in other donors a much greater proportion of the unique peptides detected was predicted to bind to one of their HLA-DRB1 alleles, with DRB1^∗^04:01, a prevalent allele in European populations, appearing to play a more dominant role in antigen presentation in both of the donors expressing this allele. The depth of immunopeptidome profiling achieved for different HLA-DR alleles correlated with protein expression levels, but certain alleles may also present a more diverse repertoire of peptides due to a preference for more common amino acids and/or ability to tolerate a greater number of different amino acid residues at key anchor positions, and/or to differences in their association with the peptide editor HLA-DM or the associated HLA-DO protein that modulates its function (Roche and Furuta, 2015).

Our analysis identified multiple S-derived peptides in the HLA-II-bound repertoire bearing glycans or other post-translational modifications. Viral envelope proteins are often heavily glycosylated and the SARS-CoV-2 S protein is no exception (Grant et al., 2020), with complex N-glycosylation stemming from 22 sites (Watanabe et al., 2020). Glycosylation of virion surface proteins acts to enhance viral infectivity and also subvert recognitions by host adaptive responses (by shielding nAb binding sites and impairing antigen processing for T cell recognition), but it is also targeted by host innate immune recognition pathways (Baum and Cobb, 2017). S protein glycosylation is carried out by the host cell glycan processing machinery, resulting in attachment of a range of oligomannosidic, complex, or hybrid structures that mimic mature surface glycoproteins of the host. We initially confirmed that these patterns were present in the intact S protein used to pulse MDDCs. Strikingly, we found that the HLA-II-bound S peptides were in contrast glycosylated at the same sites, but with glycans rich in highly processed paucimannosidic-type structures. This observation implies a significant modulation of the glycan phenotype upon internalization, processing, and presentation of the S glycoprotein in MDDCs. Paucimannosidic glycans are defined as truncated α- or β-mannosyl-terminating *N*-glycans carried by proteins expressed widely across the eukaryotic domain, but remain a poorly understood glycan class in human glycobiology and virology (Tjondro et al., 2019). We have recently reported that neutrophils (Thaysen-Andersen et al., 2015; Venkatakrishnan et al., 2020) and monocytes/macrophages (Hinneburg et al., 2020), but thus far not DCs, are paucimannose-producing cell types in the innate immune system. The paucimannosidic glycans have been proposed to be formed via the sequential trimming facilitated by the *N*-acetyl-β-hexosaminidase isoenzymes and linkage-specific α-mannosidases residing in lysosomes or lysosomal-like compartments (Tjondro et al., 2019). Supporting our data suggesting an extensive DC-driven glycan remodeling ahead of viral glycopeptide presentation, *N*-acetyl-β-hexosaminidase and α-mannosidase, and several other hydrolytic enzymes (e.g., cathepsin D), are known to be abundantly expressed and highly active in MHC class II processing compartments (MIICs) (Lankar et al., 2002). Furthermore, MHC class II immunopeptides carrying truncated *N*-glycans have previously been reported from other cellular origins (Dengjel et al., 2005; Malaker et al., 2017). CD4^+^ T cell recognition of glycosylated peptides has been reported in rheumatoid arthritis (O-linked) (Michaëlsson et al., 1996) and cancer (N-linked) (Housseau et al., 2001), and CD4^+^ peptides in the melanoma antigen tyrosinase require the presence of N-linked glycosylation to elicit a T cell response (Housseau et al., 2001). A recent study also showed that immunization of mice with a recombinant human immunodeficiency virus type 1 (HIV-1) envelope (Env) glycoprotein immunogen elicits CD4^+^ T cell responses to a glycopeptide epitope that provide help for induction of Env-specific antibody responses (Sun et al., 2020), suggesting that glycopeptide-targeting CD4^+^ T cell responses may constitute an important and under-studied component of the immune response elicited following infection or vaccination.

Further investigation of post-translational modifications in HLA-II-bound S peptides revealed prevalent cysteine modifications. Specifically, we observed cysteinylation and glutathionylation of C479 and C432, a cysteine pair that form two key disulphide bonds in the RBD (Lan et al., 2020). Free cysteines are highly reactive and during denaturation can readily become oxidized depending on the surrounding environment (Trujillo et al., 2014). The origin of cysteinylation and glutathionylation in HLA-II peptides is uncertain, and reactions could occur within the endosome or extracellular medium with free cysteine or glutathione, depending on where the peptides are loaded onto MHC molecules (Jensen, 1991). The existence of cysteine-modified viral epitopes has been explored previously for both class I (Meadows et al., 1997; Chen et al., 1999) and class II (Haque et al., 2001) epitopes, and their presentation in the immunopeptidome is allele and context dependent (Trujillo et al., 2014). Biologically, cysteine modifications potentially reflect the redox status of the cell (Trujillo et al., 2014) and can alter T cell recognition of antigens in infection, vaccination, and cancer (Chen et al., 1999; Haque et al., 2001; Jensen, 1991; Meadows et al., 1997). In agreement with our observations for the SARS-CoV-2 S protein, a class I-restricted T cell epitope derived from the RBD (312–635) of the S glycoprotein recognized by CD8^+^ T cells in mice infected with mouse hepatitis virus (a murine coronavirus), was found to be S-glutathionylated (Trujillo et al., 2014). In cancer, cysteinylation of antigens can confer evasion from T cell recognition, but processing by the interferon (IFN)-γ-inducible lysosomal thiol reductase (GILT) can remove antigen cysteinylation and induce antigen processing and T cell responses in the context of melanoma (Norton and Haque, 2009). In an antigen-presenting cell loading system similar to that used here, a requirement for peptide endocytosis and processing of a spontaneously cysteinylated peptide was required to establish T cell activation but not MHC binding and presentation (Haque et al., 2001). Thus, it will be important to determine whether the cysteine-modified S peptides described herein are targeted by T cells following vaccination or during SARS-CoV-2 infection.

Other peptide modifications (deamidation of Q/N, oxidation and per-oxidation of M, and conversion of N-terminal Q to pyroglutamic acid) were identified in our study. Such modifications are commonly observed in mass spectrometry experiments, and their abundance in the sample can be altered during the analytical processing of the sample. Oxidation of methionine has been shown to occur during electrospray ionization (Morand et al., 1993). Deamidation of glutamine and asparagine can occur spontaneously at physiological pH and temperature, and cyclization of N-terminal glutamine to pyroglutamic acid is initiated in mild acidic conditions (Yang and Zubarev, 2010). Therefore, the extent of peptide modification may depend on sample storage, processing conditions, and acquisition parameters.

Notably, the RBM, an area of the RBD important for interacting with the host receptor ACE2 (Lan et al., 2020), was found to be a HLA-DR-binding peptide-rich region, with presentation of peptides derived from amino acids 457–485 of the SARS-CoV-2 spike protein being detected in all of the HLA-diverse donors studied. Analysis of the T cell responses elicited following vaccination of mice with recombinant DNA (rDNA)-based vectors encoding the S proteins from both SARS and SARS-CoV-2 has shown that the epitopes targeted by CD4^+^ T cells include a site in the RBD that encompasses the RBM (Yang et al., 2004; Smith et al., 2020), suggesting that peptides derived from this region may be presented in a cross-species manner. Interestingly, CD4^+^ T cell responses to an epitope at amino acids 449–461 in the SARS-CoV spike (equivalent to, although having a number of sequence differences from amino acids 462–474 of the SARS-CoV-2 spike protein) were detected in healthy donors not exposed to SARS-CoV (or SARS-CoV-2) (Yang et al., 2009), indicating that RBM-derived peptides from diverse coronaviruses infecting humans may similarly be presented with HLA-II. More work is needed to determine the impact of sequence diversity on the HLA-II-binding affinity of RBM-derived peptides and also to explore the inter-virus cross-reactivity of RBM-targeting CD4^+^ T cells, but our findings highlight this as a putatively immunogenic region worthy of further study.

Our analysis also revealed promiscuous presentation of peptides derived from several sites in the N-terminal domain of S1, and a number of sites in S2. Abundantly presented sites in S2 included regions around the fusion peptide, heptapeptide repeat sequence 1 and connector domain. Given that the S2 proteins of SARS-CoV-2 and other human coronaviruses exhibit much greater sequence conservation than S1 (Lei and Zhang, 2020), the S2 peptides identified here have potential for targeting by T cell responses capable of cross-recognizing emerging SARS-CoV-2 variants (as well as other human coronaviruses), suggesting the utility of tailoring future vaccine designs to elicit strong responses to these sites.

Comparison of our HLA-II-bound S peptide profiling dataset to the SARS-CoV-2 S peptides identified as being recognized by CD4^+^ T cell responses induced in HLA-II diverse individuals infected with SARS-CoV-2 and/or seasonal coronaviruses in four recent studies (Mateus et al., 2020; Nelde et al., 2021; Peng et al., 2020; Tarke et al., 2021) revealed that 67% of the amino acids within the T cell epitope-containing long peptides overlapped with the sequence of HLA-II-bound peptides presented by spike protein-pulsed DCs. Due to the imprecise nature of the T cell epitope mapping performed, this value provides an underestimate of the proportion of the T cell-targeted sequences identified within the HLA-II-bound spike peptide repertoire defined here, but the enrichment of T cell-targeted peptides within our dataset supports the utility of our immunopeptidome profiling approach for T cell epitope identification. T cell-targeted peptides not detected in the immunopeptidome may be presented by HLA alleles whose peptide repertoires were not profiled here, emphasizing the importance of interrogation of peptide presentation by a breadth of diverse HLA-II alleles (including HLA-DQ alleles, which were not analyzed in the current study) for more comprehensive epitope identification. Importantly, HLA-II-bound peptide sequencing also has the capacity to identify peptides that are not highly immunogenic in natural infection, but to which responses could be elicited by vaccination to increase the breadth of epitopes targeted and/or enable recognition of more conserved viral sequences and facilitate cross-targeting of viral variants and related viruses. Interestingly, while 78% of the amino acids within S1-derived HLA-II-bound peptides overlapped with residues in peptides that were found to be targeted by CD4^+^ T cell responses in SARS-CoV-2 convalescent patients in the study by Tarke et al., 2021, much less T cell targeting of S2-derived peptides (53%) was detected in infected individuals. This suggests an opportunity for vaccine design to elicit more S2-focused responses, which may have a greater cross-protective potential than the heavily S1-biased responses induced during natural SARS-CoV-2 infection.

In summary, our data provide a detailed map of HLA-II-binding peptides in the SARS-CoV-2 S protein that will facilitate the analysis of CD4^+^ T cell responses to both “conventional” and novel post-translationally modified epitopes in this important viral target protein. In addition to the utility of our findings in dissection of pre-existing and post-infection responses to the SARS-CoV-2 S protein and their impact on infection outcome, our results also have application in the monitoring of vaccine-elicited immune responses and cross-comparison of the CD4^+^ T(fh) responses elicited by different immunogens, vaccine platforms, and immunization regimes. Furthermore, they have important implications for the design of vaccines aiming to target immune responses to specific sites on the S glycoprotein, e.g., indicating the potential for RBD vaccines to elicit CD4^+^ T(fh) responses in HLA-diverse vaccine recipients, suggesting an opportunity for targeting of relatively conserved peptides in S2 to which immunodominant responses are not elicited in natural SARS-CoV-2 infection, and highlighting regions of the spike protein that may be poor sources of class II epitopes, where linkage to exogenous CD4^+^ T(fh) epitopes such as broadly presented peptides from tetanus toxoid may be advantageous in future vaccine design.

### Limitations of study

In the current study, the repertoire of peptides presented with HLA-II by S protein-pulsed MDDCs derived from 5 HLA-DRB1-heterozygous donors expressing a total of 9 different HLA-DRB1 alleles was profiled using a workflow focused primarily on identification of HLA-DR-bound peptides, which also provided some insight into the HLA-DP-bound peptide repertoire. Limitations included the number of donors analyzed and diversity of HLA-II alleles they expressed, and the number of MDDCs it was possible to generate from each donor, which restricted the quantity of HLA-II available for peptide profiling. In the future, a more comprehensive map of the S protein-derived HLA-II-bound peptide repertoire could be generated by employing MDDCs from a larger number of donors selected to express HLA-DR, -DP, and -DQ alleles covering a higher proportion of global HLA-II diversity and obtaining more cells from each donor so that sufficient HLA-II was available to achieve an in-depth profiling of HLA-DR, -DP, and -DQ-bound peptides.

Note that while this manuscript was in revision, a mass-spectrometry-based analysis of HLA-II-bound SARS-CoV-2 S peptides presented on S protein-pulsed MDDCs was reported by Knierman et al. (2020), findings from which are consistent with those of the current study (although HLA-II-bound glycopeptides were not analyzed).

## STAR★Methods

### Key resources table

**Table:** 

REAGENT OR RESOURCE	SOURCE	IDENTIFIER
**Antibodies**		

Anti-HLA-DR PerCP-Cy5.5 (clone: L243)	BioLegend	307630; RRID: AB_893567
Anti-HLA-I Pacific Blue (clone: W6/32)	BioLegend	311417; RRID: AB_493668
Anti-DEC-205-BV785 (clone: MMRI-7)	BD Biosciences	744061; RRID: AB_2741964
Anti-DC-SIGN-PE-Cy7 (clone: 9E9A8)	BioLegend	330113; RRID: AB_10719952
Anti-CD86-APC (clone: 2331)	BD Biosciences	555660;RRID: AB_2646666
Anti-CD83-FITC (clone: HB15e)	BioLegend	305305; RRID:AB_314513
Anti-CD80-BV650 (clone: 2D10)	BioLegend	305227; RRID: AB_11203545
Anti-CD40-PE (clone: 5C3)	BioLegend	334308; RRID: AB_1186038
Anti-CD11c-APC/Fire 750 (clone: S-HCL-3)	BioLegend	371509; RRID:AB_2650792
W6/32	ATCC	HB-95
L243	ATCC	HB-55
B7.21	Gift from Prof. Anthony Purcell, Monash University, Australia. anthony.purcell@monash.edu	na

**Biological samples**		

Frozen PBMCs	NHS Blood and Transplant Service, Oxford	https://www.ouh.nhs.uk/haematology/services/#nhsbt

**Chemicals, peptides, and recombinant proteins**		

Trypsin	Promega	V5111
Elastase	Promega	V1891
PNGaseF	New England Biolabs	P0704S
LIVE/DEAD Fixable Aqua Dead Cell Stain Kit	Thermo Fisher Scientific	L34957

**Deposited data**		

Mass spectrometry peaks lists and sequence annotations	This study; PRIDE Database https://www.ebi.ac.uk/pride/	PXD021672
Raw mass spectrometry files	This study; PRIDE Database https://www.ebi.ac.uk/pride/	PXD021672

Supplementary Tables 1-4	This study; Mendeley Data: https://doi.org/10.17632/mk3xmv46n8.1	10.17632/mk3xmv46n8.2
**Experimental models: Cell lines**		

FreeStyle293F	Thermo Fisher Scientific	R79007

**Recombinant DNA**		

SARS-CoV-2 expression plasmid	Henderson et al., 2020; Wrapp et al., 2020	https://www.ncbi.nlm.nih.gov/nuccore/MN908947

**Software and algorithms**		

IEDB	Vita et al., 2019	https://www.iedb.org
Peaks X	Bioinformatic solutions	https://www.bioinfor.com/peaks-studio/
R	The R Project for Statistical Computing	https://www.r-project.org/
Byonic	PROTEIN METRICS	https://proteinmetrics.com/byos/
NetMHCIIpan 4.1	DTU Health Tech	http://www.cbs.dtu.dk/services/NetMHCIIpan/

### Resource availability

#### Lead contact

Further information and requests for resources and reagents should be directed to and will be fulfilled by the Lead Contact, Nicola Ternette, nicola.ternette@ndm.ox.ac.uk.

#### Materials availability

This study did not generate new unique reagents

#### Data and code availability

The mass spectrometry proteomics data have been deposited to the ProteomeXchange Consortium via the PRIDE (Perez-Riverol et al., 2019) partner repository with the dataset identifier PRIDE: PXD021672 and 10.6019/PXD021672. Additional Supplemental Items are available from Mendeley Data: https://doi.org/10.17632/mk3xmv46n8.1.

### Experimental model and subject details

Leukocyte cones obtained with appropriate ethical approval from healthy human donors who provided written informed consent were purchased from the NHS Blood and Transplant Service, Oxford. Donor sex and age is unknown. All work was compliant with institutional guidelines. Mononuclear cells were isolated from leukocyte cones by separation on a Histopaque-1077 gradient then stored in liquid nitrogen. Genomic DNA was extracted from cells using the QIAamp DNA Mini Kit (QIAGEN), then the HLA-DRB1, -DPB1, and -DQB1 loci were sequenced at the OUH Transplant Laboratory by Sanger sequencing using an ABI-3730 DNA analyzer.

### Method details

#### Protein expression and purification

The SARS-CoV-2 ectodomain constructs were produced and purified as described previously (Henderson et al., 2020; Wrapp et al., 2020). Briefly, the expression construct included residues 1−1208 of the SARS-CoV-2 S (GenBank: MN908947) with proline substitutions at residues 986 and 987, a “GSAS” substitution at the furin cleavage site (residues 682–685), a C-terminal T4 fibritin trimerization motif, an HRV3C protease cleavage site, a TwinStrepTag and an 8XHisTag. Expression plasmids encoding the ectodomain sequence were used to transiently transfect FreeStyle293F cells using Turbo293 (SpeedBiosystems). Protein was purified on the sixth day post transfection from the filtered supernatant using StrepTactin resin (IBA), followed by size exclusion chromatography using a Superose 6 Increase 10/300 column.

#### Differentiation of monocyte-derived DCs (MDDCs)

To differentiate MDDCs, mononuclear cells were thawed and plated at 10^6^ cells per cm^2^ in 20 mL RAB5 (RPMI with 5% human serum AB (Sigma), 2 mM L-alanyl-L-glutamine, 10mM HEPES, 50 units/ml penicillin, 50 μg/ml streptomycin) per 175 cm^2^ flask for 2 hours at 37°C. Non-adherent cells were removed by three gentle PBS washes. The remaining adherent cells were cultured in RAB5 containing 300 IU/ml IL-4 and 100 IU/ml GM-CSF for 6 days. MDDCs were harvested on day 6 by incubation in cell dissociation solution (Sigma) followed by gentle scraping. Cells were resuspended in AIM V medium (Thermo Fisher Scientific) and incubated with 0.5 mg of recombinant SARS-CoV-2 S protein or an irrelevant viral envelope glycoprotein produced in the same way for 18 hours. Cells were then harvested by gentle scraping then washed before lysis for HLA immunopurification.

#### Flow cytometry

Following differentiation and protein pulse, MDDCs were washed and stained with antibodies specific for HLA-DR (L243, Biolegend), HLA-I (W6/32, Biolegend), DEC-205 (MMRI-7, BD Biosciences), DC-SIGN (9E9A8, Biolegend), CD86 (2331, BD Biosciences), CD83 (HB15e, Biolegend), CD80 (2D10, Biolegend), CD40 (SC3, Biolegend) and CD11c (S-HCL-3, Biolegend) for 20 minutes at room temperature. Cells were then washed and fixed in 4% PFA. Staining data were acquired on a LSRFortessa and analysis was performed on Flowjo software version 10.6.2.

#### Enzymatic digestion of SARS-CoV-2 S protein

An equivalent of 5 ug of SARS-CoV-2 S protein was reduced by incubation with 5 mM DTT for 30 minutes. Reduced S was incubated for 30 minutes with 20 mM iodoacetamide (IA) followed by addition of DTT to 20 mM to react with residual IA. 0.2 μg of trypsin or elastase was added per 5 μg of CoV2 S and incubated for 16 hours at 37°C. Sample clean-up was performed with a C18 column (Waters Oasis SPE kit).

#### PNGaseF deglycosylation of digested SARS-CoV-2 S protein

Digested CoV2 S protein was dried in a SpeedVac and resuspended in 10 ul 20mM sodium phosphate pH 7.5. Samples were divided equally, dried in a SpeedVac and then resuspended in ^18^O-water (Sigma-Aldrich, 97%) containing 200 U PNGaseF (New England Biolabs) or buffer only. The protein was digested for 16 hours at 37°C. Sample clean-up was performed with a C18 column (Waters Oasis SPE kit) and peptides were dried in a SpeedVac.

#### Production and purification of HLA-specific antibodies

Hybridoma cells (clones W6/32, L243, B721) were cultured in a CELLine CL 1000 Bioreactor (Integra) as described in (31). B721 was kindly supplied by Prof. Anthony Purcell, Monash University, Melbourne, Australia. Briefly, cells were cultured in serum-free Hybridoma-SFM medium (GIBCO) supplemented with hybridoma mix (2,800 mg/l of d-glucose, 2,300 mg/l of peptone, 2 mM l-glutamine, 1% penicillin/streptomycin, 1% non-essential amino acids, 0.00017% 2-mercaptoethanol). Supernatant containing antibody was harvested and stored at −20. To purify antibodies, supernatants were thawed then centrifuged at 2,500 × g for 25 min at 4°C, filtered (0.2 μm SteriCup Filter (Millipore)) and incubated with protein A resin (PAS) (Expedeon) for 18 hours at 4°C. Antibody-resin complexes were then collected by gravity flow through chromatography columns, washed with 20 mL of PBS, and eluted with 5 mL 100 mM glycine pH 3.0. pH was adjusted to pH 7.4 using 1 M Tris pH 9.5 and antibodies were buffer exchanged into PBS and concentrated with a 5 kDa molecular weight cut-off ultrafiltration device (Millipore).

#### Preparation of immunoresin

Cross-linking of purified antibodies was performed as follows: 3 mg of antibody per 0.5 mL PAS was incubated for 1 h at 4°C then washed with 15 mL borate buffer (0.05 M boric acid, 0.05 M KCl, 4 mM NaOH, pH 8.0), 15 mL 0.2 M triethanolamine pH 8.2 and cross-linked to PAS with 15 mL of 40 mM dimethyl pimelimidate in 0.2 M triethanolamine pH 8.3. After 1 h, crosslinking was terminated by addition of 15 mL of 0.2 M Tris pH 8.0, unbound antibody was removed by washing with 15 mL citrate buffer pH 3.0 and three washes with 15 mL PBS.

#### HLA complex immunoprecipitation (IP) and peptide purification

Harvest MDDCs were washed in PBS then lysed by mixing for 45 minutes with 3 mL of lysis buffer (0.5% (v/v) IGEPAL 630, 50 mM Tris pH 8.0, 150 mM NaCl and 1 tablet cOmplete Protease Inhibitor Cocktail EDTA-free (Roche) per 10 mL buffer) at 4°C. Lysate clarification was achieved by centrifugation at 3,000 x g for 10 mins followed by a 20,000 × g spin step for 15 mins at 4°C. 3 mg of W6/32 antibody-PAS was incubated with lysate for at least 5 h at 4°C with gentle rotation. Resin was collected by gravity flow and flow-through lysate was sequentially incubated with L243- and then B721-PAS. Antibody-resin-HLA complexes were washed with 15 mL of 0.005% IGEPAL, 50 mM Tris pH 8.0, 150 mM NaCl, 5 mM EDTA, 15 mL of 50 mM Tris pH 8.0, 150 mM NaCl, 15 mL of 50 mM Tris pH 8.0, 450 mM NaCl, and 15 mL of 50 mM Tris pH 8.0. 3 mL of 10% acetic acid was used to elute bound HLA complexes from the PAS-antibody resin, which were then dried by vacuum centrifugation. Eluted peptides were dissolved in loading buffer (0.1% (v/v) trifluoroacetic acid (TFA), 1%(v/v) acetonitrile in water), and then injected by a Ultimate 3000 HPLC system (Thermo Scientific) and separated across a 4.6 mm × 50 mm ProSwift RP-1S column (Thermo Scientific). Peptides were eluted using a 1 ml/min gradient over 5 min from 1%–35% Acetonitrile/0.1% TFA and 15 fractions were collected every 30 s. Peptide fractions 1-10 were combined into odd and even fractions then dried.

#### Enzymatic digestion of HLA proteins in immunoprecipitates for LC-MS analysis

HPLC fractions 11-15 containing the protein portion of the HLA complex immunoprecipitation (IP) were dissolved in 5% (w/v) SDS and processed using the S-Trap micro protocol (ProtiFi). Briefly, samples were reduced and alkylated as described above, acidified to 12% (v/v) phosphoric acid, applied to the S-trap column, washed with 50 mM TEAB, 90% (v/v) methanol, before being digested with 1 ug of sequencing grade trypsin. Peptides were eluted with 50% (v/v) acetonitrile, 0.1% (v/v) Formic acid, dried and resuspended in 0.1% TFA (v/v), 1% (v) acetonitrile.

#### Liquid chromatography - mass spectrometric (LC-MS) acquisition

HPLC fractions were dissolved in loading solvent, and analyzed by an Ultimate 3000 HPLC system coupled to a high field Q-Exactive (HFX) Orbitrap mass spectrometer (Thermo Scientific). Peptides were initially trapped in loading solvent, before RP separation with a 60 min linear acetonitrile in water gradient of 2-25 or 35% (HLA-I/HLA-II and trypsin analyses) across a 75 μm × 50 cm PepMap RSLC C18 EasySpray column (Thermo Scientific) at a flow rate of 250 nl/min. Gradient solvents contained additional 1%(v/v) DMSO and 0.1%(v/v) formic acid. An EasySpray source was used to ionise peptides at 2000 V, and peptide ions were introduced to the MS at an on transfer tube temperature of 250°C. Ions were analyzed by data-dependent acquisition. Initially a full-MS1 scan (120,000 resolution, 60 ms accumulation time, AGC 3X10^6^) was followed by 20 data-dependent MS2 scans (60,000 resolution, 120 ms accumulation time, AGC 5X10^5^), with an isolation width of 1.6 m/z and normalized HCD energy of 25%. For HLA-II charge states of 2–4 were selected for fragmentation, for HLA-I 1+ ions were also included. Dynamic exclusion was set for 30 s. For enzymatic digests of S protein and immunocomplexes normalized HCD was increased to 28%, only 2-4 charge states were acquired.

#### LC-MS data analysis

Raw data files were analyzed in PEAKS X software (Bioinformatic Solutions) using a protein sequence fasta file containing 20,606 reviewed human Uniprot entries downloaded on 24/05/2018, supplemented with the sequence for SARS-CoV-2 full-length S protein cloned. No enzyme specificity was set, peptide mass error tolerances were set at 5 ppm for precursors and 0.03 Da for MS2 fragments. Additionally, post translational modifications were identified utilizing the Peaks PTM inbuilt *de novo*-led search for 303 common modifications. A 1% false discovery rate (FDR) was calculated using decoy database search built into Peak data plotting. Data manipulation was performed in R and excel. NetMHCII pan 4.0 (Reynisson et al., 2020) (http://www.cbs.dtu.dk/services/) was installed locally and utilized to define binding (rank score cut of 10). Sequence logos were generated by Seq2logo2.0. Venn diagrams and UpsetR plots were created using the online http://www.interactivenn.net/index2.html (Heberle et al., 2015) and UpsetR program in R. Clustering of sequences was done by GibbsCluster2.0 with defaults for MHC class II ligands and 1–5 clusters, the most obvious 1-2 clusters were selected. Peptides were aligned into nested sets using Immune Epitope Database (IEDB) Epitope Cluster Analysis (Dhanda et al., 2018) tool http://tools.iedb.org/cluster/ and positions in proteins were determined by http://tools.iedb.org/immunomebrowser/. A python script was written to calculate the frequency of amino acids across the full length S protein. Jalview 2.11.1.0 (Waterhouse et al., 2009) was used for multisequence alignment with MAFFT (Multiple Alignment using Fast Fourier Transform) with default settings. Alignments were visualized in Jalview and coloring amino acids by property based on CLustal_X definitions or identity. All graphs were plotted in R or Excel.

#### Glyco-site and glycopeptide analysis

Raw data for the de-*N*-glycosylated samples were analyzed as described above with the following modifications. A standard PeaksDB search was utilized with additional variable post translational modifications set for ^18^O labeling (C-terminal +2.00 Da), ^18^O deamidation (NQ, +2.99 Da), deamidation (NQ, −0.98 Da) and oxidation (M, +15.99 Da) and a fixed modification for carboxyamidomethylation (C, +57.02). Each site was checked manually for over-lapping peptides with consistent heavy deamidation present. Glycopeptides were identified by Byonic v3.8.13 (Protein Metrics, CA, USA). Spectra were extracted and searched against the same fasta file as above, but with unspecific cleavage sites and allowing unlimited missed cleavages. A library of 132 possible *N*-glycan compositions was utilized in a ‘rare’ variable modification search strategy. A fixed modification of carboxyamidomethylation was included for digested S. Data was filtered at a 1% protein FDR resulting in a 1.38% peptide FDR in the combined dataset, glycopeptides were further filtered to ensure that oxonium ions were present in the MS2 spectrum and that scores were above 150 as described (Lee et al., 2016). All reported glycopeptides were manually checked. Filtering and spectral counting was performed in Byologic v3.8.11, spectra with the same precursor mass and peptide identity was grouped and compositions determined as per the library.

### Quantification and statistical analysis

Quantitative proteomics data analysis was performed in Maxquant v 1.6.1.0, utilizing the same human sequences used above except all HLA proteins were substituted by the sequence of the HLA-II found in the donor, downloaded from https://www.ebi.ac.uk/ipd/imgt/hla/. Variable modifications were set to N-terminal (M+ 42.01) acetylation and oxidation (M, +15.99 Da), a fixed modification for carbamidomethylation (C, +57.02) was also set and only peptides and proteins passing 1% FDR were reported. IBAQ values were determined using only unique peptides, enabling allele-specific, relative quantitation.

## Supplemental information

Document S1. Figures S1–S7

Table S1. Supplementary data for immunopeptidomic data analysis, providing peptide identification metrics, nested clusters, and NetMHCIIpan predictions for all peptides identified by the Peaks search engine for sequences that map to S protein in all donors

Table S2. Supplementary data for elastase digestion of purified S protein and treatment with PNGnase F in the presence of H2O18, providing site, modification, peptide identification score, and occupancy

Table S3. Supplementary data for elastase digestion of purified S protein, providing glycopeptide identification metrics from Byonic search engine for glycopeptide sequences that map to S protein

Table S4. Supplementary data for immunopeptidomic samples, providing peptide identification metrics, nested clusters, and NetMHCIIpan predictions for all peptides identified by Byonic search engine for glycopeptide sequences that map to S protein engine in all donors

Table S5. Supplementary data for immunopeptidomic samples, providing peptide identification metrics, nested clusters, and NetMHCIIpan predictions for all peptides identified by Byonic search engine for glycopeptide sequences that map to S protein engine in all donors

Document S2. Article plus supplemental information
