# Supplementary material for: Mapping the SARS-CoV-2 spike glycoprotein-derived peptidome presented by HLA class II on dendritic cells
Source: Cell Rep. 2021 May 13;35(8):109179. doi: 10.1016/j.celrep.2021.109179 (PMC8116342; doi:10.1016/j.celrep.2021.109179)
Supplement: Document S1. Figures S1–S7 [file mmc1.pdf]

Cell Reports, Volume *35*

Supplemental information

Mapping the SARS-CoV-2 spike glycoprotein-derived peptidome presented by HLA class II on dendritic cells

Robert Parker, Thomas Partridge, Catherine Wormald, Rebeca Kawahara, Victoria Stalls, Maria Aggelakopoulou, Jimmy Parker, Rebecca Powell Doherty, Yoanna Ariosa Morejon, Esther Lee, Kevin Saunders, Barton F. Haynes, Priyamvada Acharya, Morten Thaysen-Andersen, Persephone Borrow, and Nicola Ternette

A

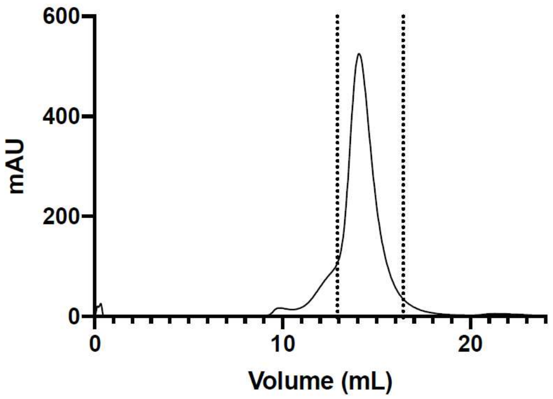

B

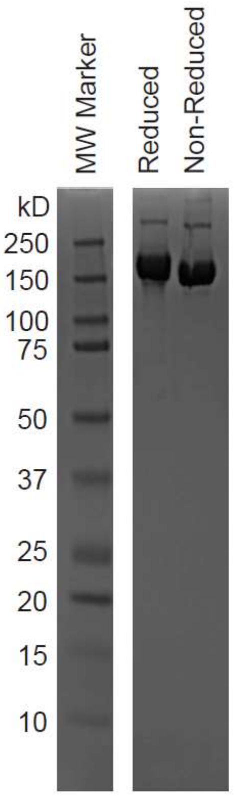

C

Furin cleavage site

SPIKE_SARS2 651 IGAEHVNNSYECDIPIGAGICASYQTQTNSPRRARSVASQSIIAYTMSLG
Recombinant SPIKE_SARS2* 651 IGAEHVNNSYECDIPIGAGICASYQTQTNSPGSASSVASQSIIAYTMSLG

SPIKE_SARS2 951 VVNQNAQALNTLVKQLSSNFGAISSVLNDILSRLDKVEAEVQIDRLITGR
Recombinant SPIKE_SARS2* 951 VVNQNAQALNTLVKQLSSNFGAISSVLNDILSRLDPPEAEVQIDRLITGR

Figure S1, related to STAR Methods. Production, purification, and sequence variation of the SARS-CoV-2 S ectodomain. A. Size exclusion chromatography profile of the SARS-CoV-2 S protein that was purified using the C-terminal TwinStrep tags. The S protein was run on a Superose 6 Increase 10/300 column. The dotted lines indicate the portion of the peak that was collected and used for this study. B. SDS-PAGE gel of the purified S protein with lanes from left to right showing molecular weight marker, S protein run under reducing conditions and S protein run under non-reducing conditions. C. Pairwise sequence alignment of regions of the S protein sequence (P0DTC2, SPIKE_SARS2, displayed as top sequence) and corresponding regions of the sequence of the recombinant S protein employed in this study (bottom sequence). Peptides found in the immunopeptidome are indicated in red text, and the sites where the amino acid sequence of the recombinant protein differs from the Uniprot S sequence are highlighted in yellow.

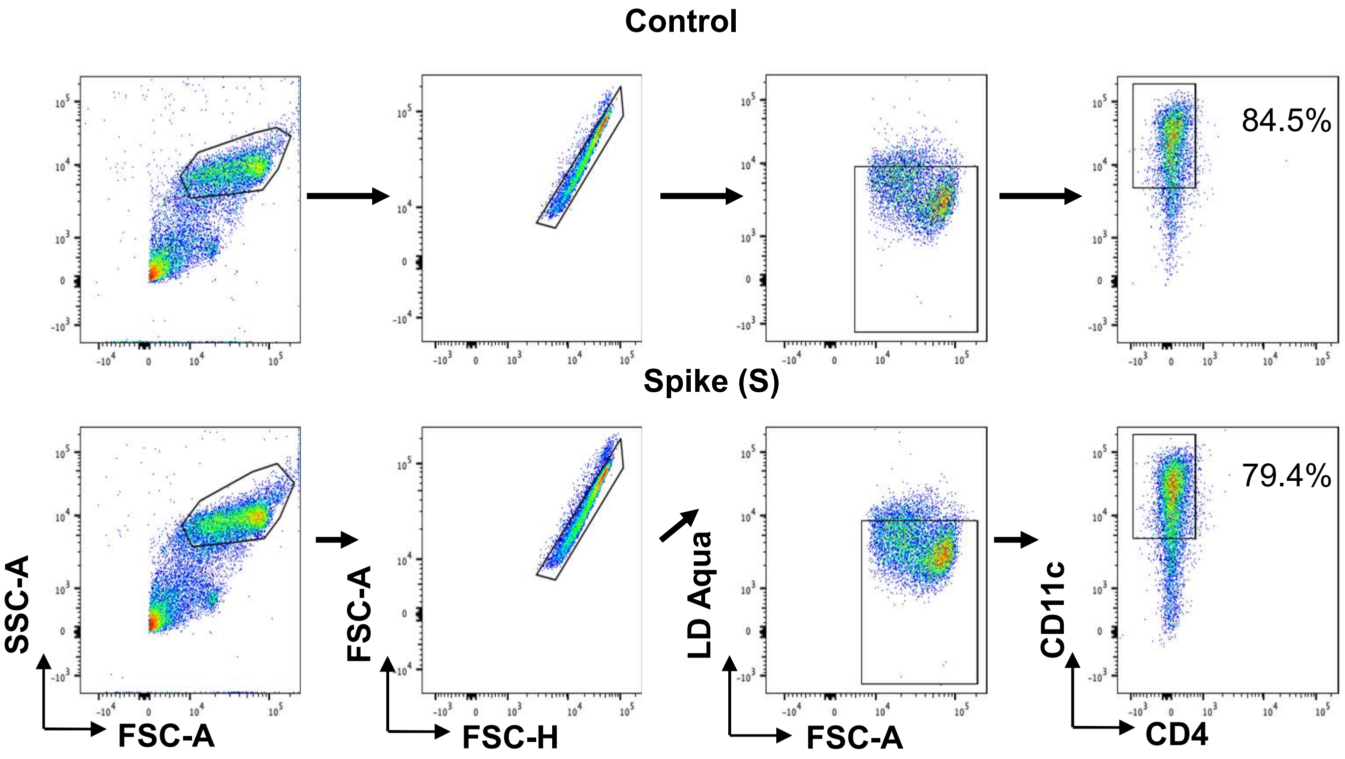

Figure S2, Related to Figure 1. Gating strategy for flow cytometry analysis of MDDCs. The dotplots show examples of the gates applied to identify MDDCs in samples from one representative donor after pulsing with S (top) or control protein (bottom). Serial gates were applied to identify large mononuclear cells, singlets, live cells and CD11c+ MDDCs (the percentage of which within the live mononuclear cell population is indicated).

A

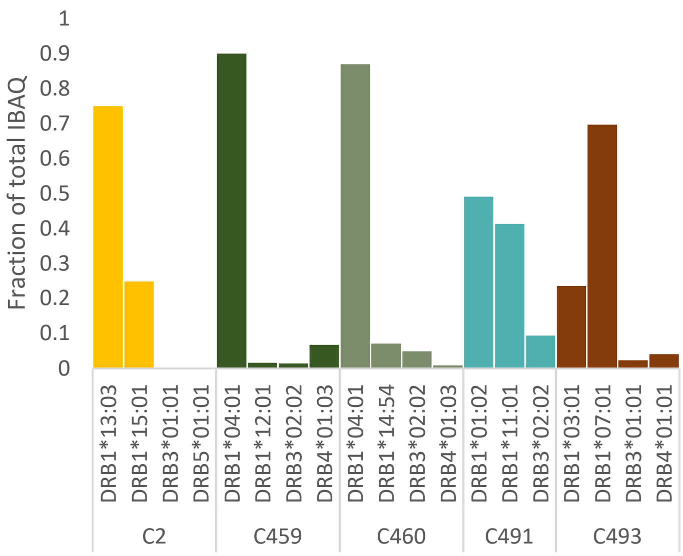

B

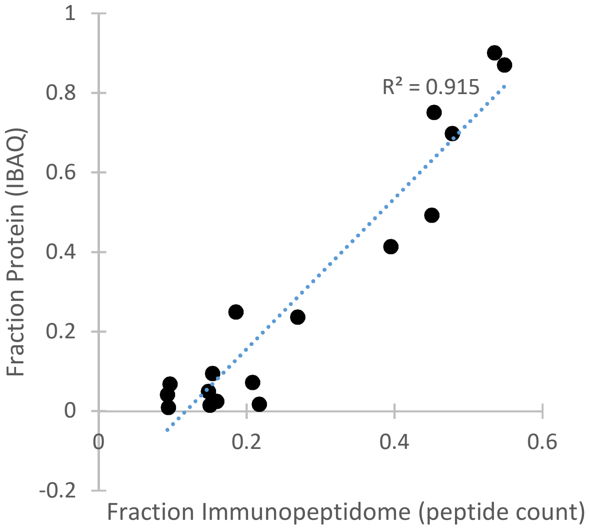

Figure S3, related to Figures 2 and 3. A. Bar plot of proportion of IBAQ protein intensity for HLA-DR1-5 identified by quantitative proteomic analysis of immunoprecipitates, stratified by allele for each donor. B. Scatter plot of the proportion of IBAQ protein intensity on the y axis and the proportion of total predicted HLA-DR1-5 binder peptides identified for each allele in the relevant donor on the x axis.

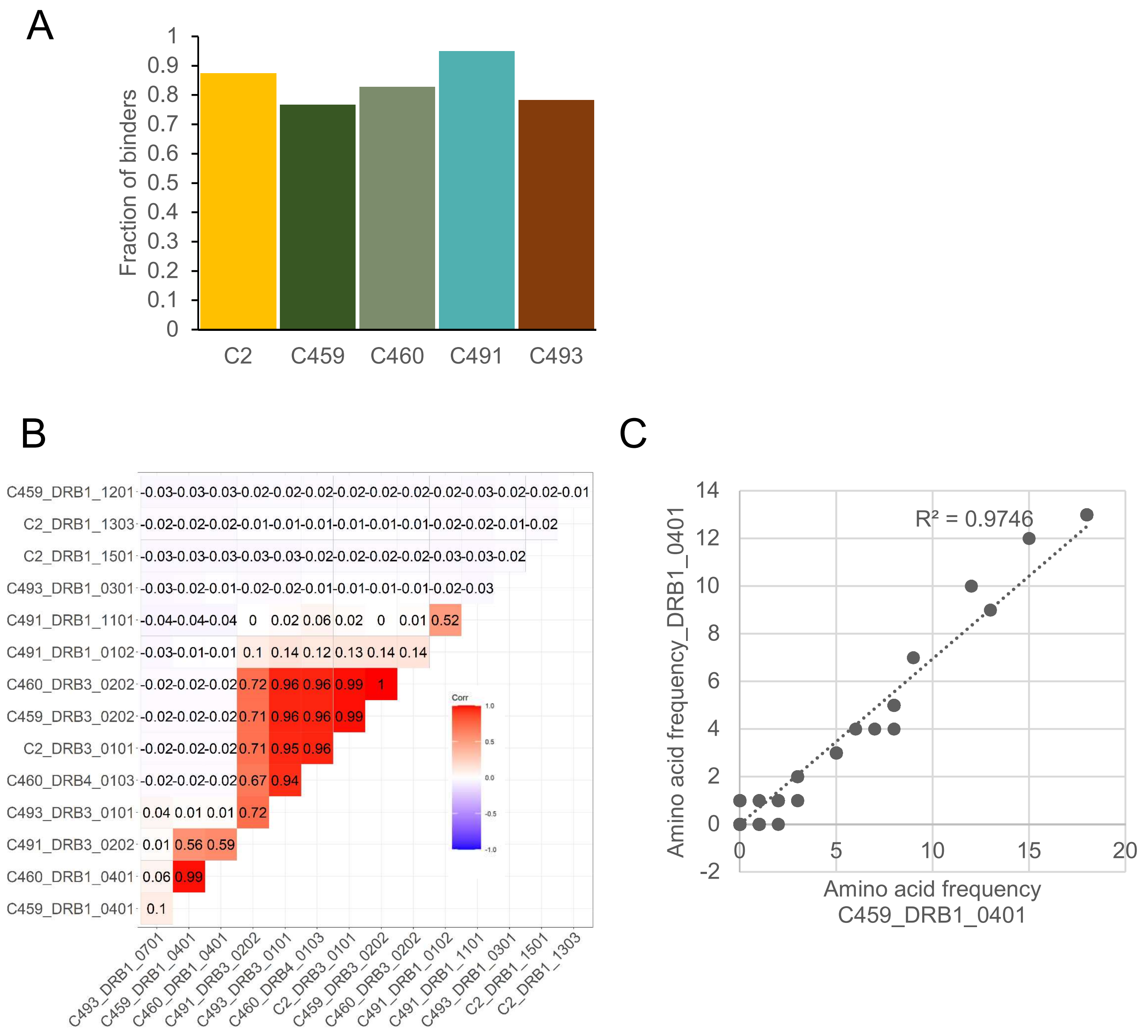

Figure S4, related to Figures 2, 3 and S5. Binding prediction and sequence correlation for S peptides identified in the HLA-DR pulldown. A. Proportion of HLA-DR peptide sequences predicted to bind to each donor's HLA-DR alleles by NetMHCIIpan4.0. B. Correlation matrix indicating the concordance in the sequence of the peptides predicted to bind to the HLA-DR alleles indicated in each donor. Pearson correlation coefficients of the frequency with which each amino acid in the S protein is represented in the peptides predicted to bind the indicated donor DR alleles were calculated and R squared values are shown; the direction and magnitude of the correlation is indicated by colour. C. Scatter plot of the frequency (number of times) with which each individual amino acid position within S is represented in all eluted S peptides predicted to bind to HLA-DRB1*04:01 in the two donors who shared this allele. Residues within S which were not embodied by peptides predicted to bind HLA-DRB1*04:01 are not plotted.

A

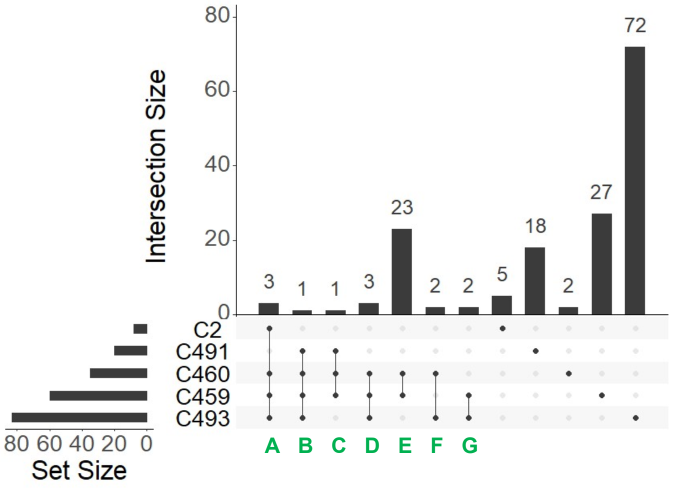

B

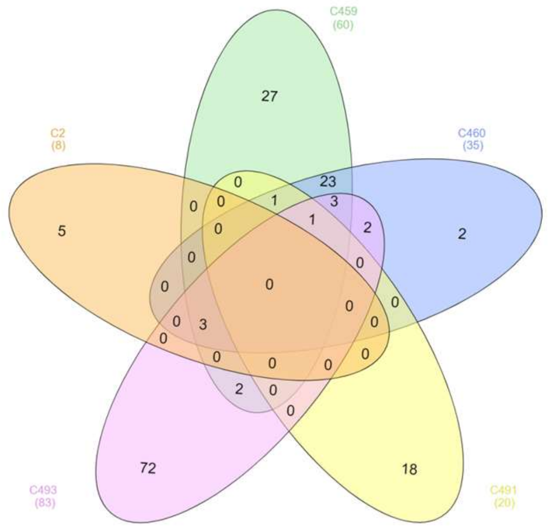

C

A

C2 and C459 and C460 and C493:

RGVYYPDK
SNLKPFERDISTEIYQA
SNLKPFERDISTEIYQ-

B

C459 and C460 and C491 and C493:

SNLKPFERDISTEIYQAG

C

C459 and C460 and C491:

RGVYYPDKVFRSSVL

D

C459 and C460 and C493:

RGVYYPDKVFRS
RGVYYPDKVFR-
LKPFERDISTEIYQ

Figure S5, related to Figures 2, 3 and S4. Peptide overlap between analysed donors. A. UpSet R plot showing the number and degree of co-occurrence of the same S peptide sequences in the HLA-DR-bound immunopeptidomes of different donors. B. Venn diagram representing the data from (A), demonstrating that there were no S peptides that were detected in the HLA-II-bound immunopeptidomes of all donors. C. Sequences of the S peptides found to be presented in more than 3 donors.

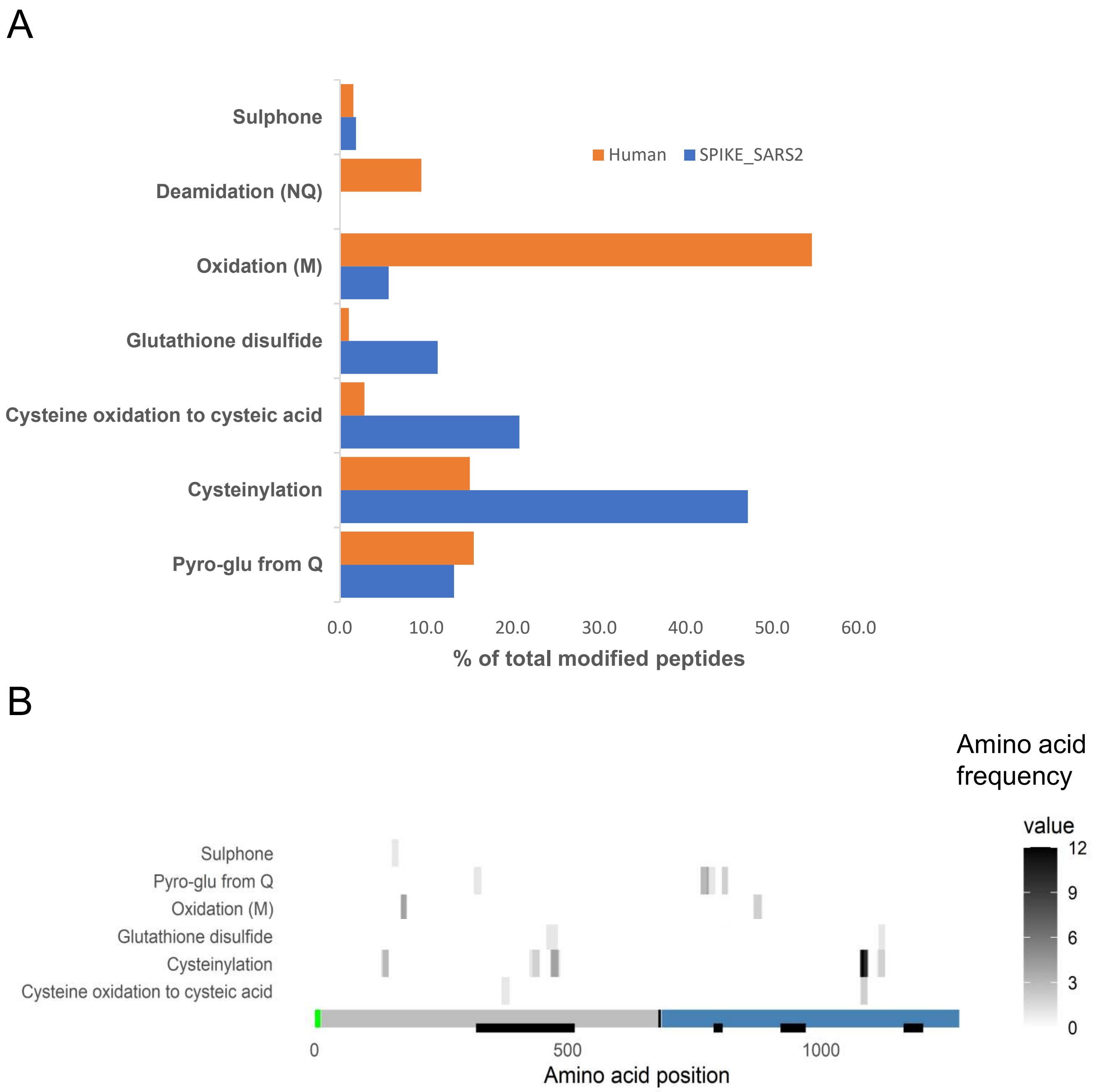

Figure S6, related to Figure 4. PTM profile of HLA-II peptides. A. Bar graph showing the most common peptide modifications detected as a proportion of all modifications reported (A-score >500), in both peptides mapping to S (blue) or mapping to human proteins (orange). B. Heatmap of positional amino acid frequency in S for the most commonly modified S peptide sequences identified in the HLA-II-bound immunopeptidome.

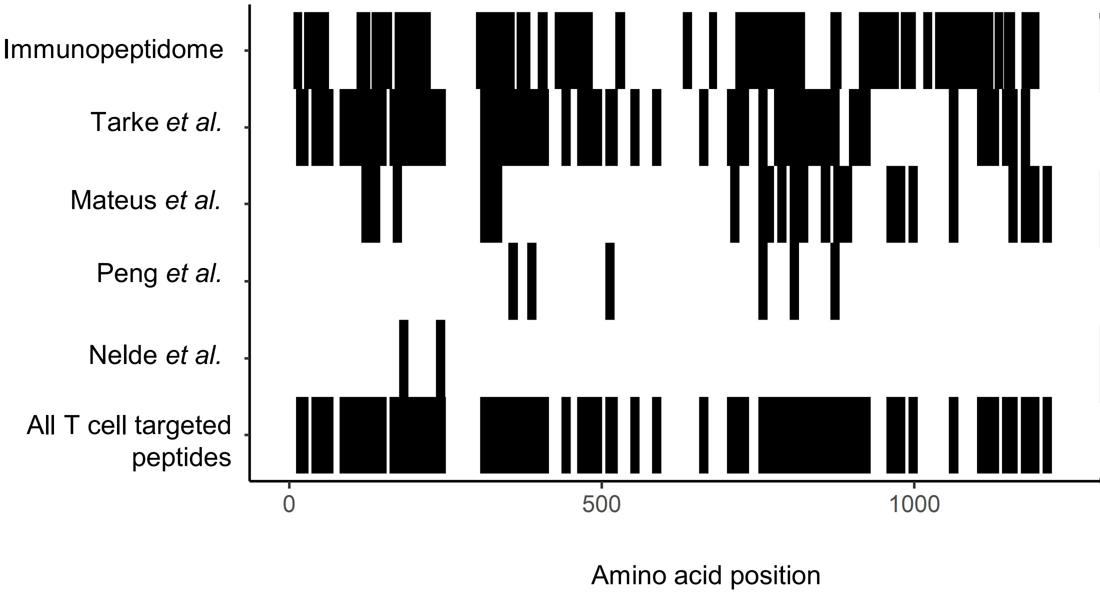

	% coverage of S	% of immunopeptidome in peptides recognised by T cells	% of T cell targeted peptides in immunopeptidome
Immunopeptidome	59.5	-	-
Tarke *et al.*	57.3	65.0	67.5
Mateus *et al.*	25.9	33.5	77.0
Peng *et al.*	7.1	8.2	68.9
Nelde *et al.*	2.4	2.0	50.0
All T cell targeted peptides	65.2	73.9	67.5

Figure S7 related to Figure 6E. Location within the spike protein sequence of peptides identified in the HLA-II-bound peptidome of spike protein-pulsed dendritic cells and peptides to which CD4+ T cell responses were detected in four epitope-mapping studies in the literature. Black bars represent the location across the S amino acid sequence (x-axis) of the peptides in each of the datasets indicated [Immunopeptidome, as profiled in the current study; T cell epitope mapping studies performed by Tarke et al (Tarke *et a*l., 2020), Mateus et al (Mateus *et al*., 2020), Peng et al (Peng *et al*., 2020), and Nelde et al (Nelde *et al*., 2020); or all four T cell epitope mapping studies combined (All T cell-targeted peptides)]. For each dataset, the % coverage of all the amino acids in the spike sequence (% coverage of S), the % coverage of amino acids within peptides identified in the HLA-II-bound spike peptide repertoire that were reported to be recognized by T cells in each study (% of immunopeptidome in peptides recognised by T cells), and the % coverage of the amino acids in each of the T cell datasets represented within the immunopeptidome (% of T cell targeted peptides in immunopeptidome) are listed.

Supplemental Tables S1-5

Table S1, relates to Figure 2 and 3: Supplementary data for immunopeptidomic data analysis, providing peptide identification metrics, nested clusters and NetMHCIIpan predictions for all peptides identified by the Peaks search engine for sequences that map to S protein in all donors.

Table S2, relates to Figure 3 and 4: Supplementary data for elastase digestion of purified S protein and treatment with PNGnase F in the presence of H2O18, providing site, modification, peptide identification score and occupancy.

Table S3, relates to Figure 4: Supplementary data for elastase digestion of purified S protein, providing glycopeptide identification metrics from Byonic search engine for glycopeptide sequences that map to S protein.

Table S4, relates to Figure 4: Supplementary data for immunopeptidomic samples, providing peptide identification metrics, nested clusters and NetMHCIIpan predictions for all peptides identified by Byonic search engine for glycopeptide sequences that map to S protein engine in all donors.

Table S5, relates to Figure 6: Supplementary data showing the spike peptides to which $CD4^{+}$ T cell responses were detected in four recent T cell epitope mapping studies (Mateus et al., 2020), (Tarke et al., 2020), (Peng et al., 2020), (Nelde et al., 2020).
